# Human health risk assessment of metal-contaminated soils in Sydney estuary catchment (Australia)

**DOI:** 10.1007/s10653-024-01898-4

**Published:** 2024-03-14

**Authors:** Gavin Birch, Xiaoyu Wang, Enfeng Liu

**Affiliations:** 1 Geocoastal Research Group, School of Geosciences, Sydney University, Sydney, NSW 2006 Australia; 2 College of Geography and Environment, Shandong Normal University, Jinan, 250358 People’s Republic of China

**Keywords:** Non-carcinogenic risk, Carcinogenic risk, Pb, Guidelines, Metals, Exposure

## Abstract

Sydney estuary catchment supports the largest city in Australia and provides essential eco-social and environmental services; however, the region has been influenced by extensive anthropogenic modification. Soil metal concentrations in the catchment had been studied previously; however, the current investigation was designed to determine the risk posed by these contaminants to human health. Soil metal concentrations were higher than observed in most global capitals and increased substantially in the south and south-east of the catchment and close to the central business district. Road-side soils and road dust contained the highest concentration of metals in the catchment. Lead in catchment soils was closely related to traffic density and sourced from the historic use of Pb in petrol. A human health assessment indicated that soil Cd, Ni and Zn posed no non-carcinogenic risk (NCR), or carcinogenic risk (CR) for children, or adults in Sydney estuary catchment and that Cu and Cr may pose minor NCR for children. Vehicle-related Pb raised the greatest human health risk in catchment soils and may pose NCR at 32% and 4.3% of sites for children and adults, respectively. Inconsistent analytical techniques used in CR and NCR evaluations produce incomparable assessments and a consistent` methodology is suggested to improve interpretation. Human health risk may well be higher than commonly calculated due to pollutants present in urban soil not being included in assessments.

**Supplementary Information:**

The online version contains supplementary material available at 10.1007/s10653-024-01898-4.

## Introduction

The Sydney estuary (50 km^2^) is a drowned, dendritic river valley 30 km long and 3 km wide in central New South Wales (NSW), Australia (Birch et al., 2021, 2022; Roy, 1983). The estuary is bound in the north by small sandy beaches and rocky headlands and large, shallow embayments lined by mangrove forests in the south (Liu, 1989). In the north Sydney estuary catchment is a high, eroded plateau and is low and undulating to the south (Birch & Lound, 2021, 2022). The small catchment (500 km^2^) is mostly developed (76.8%) and supports a high-density population (2000 people/km^2^) of 1.42 million (ABS, 2011; Johnson et al., 2017). The catchment is highly productive providing a large range of goods and services to the community (Birch et al., 2016a, b; Hoisington, 2015).

The distribution and concentration of metals in urban soils of Sydney estuary catchment have been established previously (Birch et al., 2011); however, the potential human carcinogenic risk (CR) and non-carcinogenic risk (NCR) to the urban population posed by soils have not been established. The highest soil metal concentrations were found in the oldest and most densely populated regions of the catchment and declined with distance from the central business district (CBD). The similarity in soil metal distribution and modelled deposition of atmospheric vehicular emissions (Lawrence, 2006) strongly suggested vehicular contributions were a major source of metals to catchment soils. The high concentrations and large range of metals in these soils may adversely impact human health, e.g. kidney, gastro-intestinal and nervous systems and reduce intelligence quotient by ingestion, inhalation and through skin contact (Young, 2005; Mazumdar, 2008; Cobbina et al., 2013; Zhang et al., 2012; NTP, 2012). The aims of the present study were to: (i) assess human CR and NCR posed by catchment soil; (ii) establish the major pathways of potential uptake; (iii) determine the relative risk to children and adults; (iv) and identify possible sources of metals in various land use types posing risk.

### Previous studies of soil metals in Sydney estuary catchment

The first regional study of soil in the Sydney estuary catchment established that 50% of Cu, Pb and Zn concentrations and 2.5% of Cd concentrations were above Australian and New Zealand Environment and Conservation Council and National Health and Medical Research Council (ANZECC and NH&MRC, 1992) guidelines of 300 µg/g, 200 µg/g, 60 µg/g and 3 µg/g, respectively (Markus and McBratney, 2001) (Table 1). The distance from roads explained considerable variance in total Pb (24% of variance), Zn (15% of variance), Cu (15% of variance) and Cd (13% of variance). The greatest soil metal concentrations were in highly urbanised (> 90%) catchments and in these older urban areas near major road intersections, soil health guidelines were exceeded for Cu (34% of samples), Pb (33% of samples) and Zn (56% of samples) in areas where buildings were oldest and where major roads converged. (Snowdon & Birch, 2004). Metals in soil of other highly urbanised (48%) and industrialised (19%) sub-catchments exceeded Environmental Investigation Limits for Cu (for 8% of samples), Ni (3% of samples), Pb (5% of samples) and Zn (7% of samples). Linear mixed models determined that the main drivers of contamination in catchment soils were elevation, distance from main roads, road type, landscape, population density and land use (Johnson et al., 2017). Additional modelling indicated that had Pb diminished significantly and that As and Zn had substantially increased in some landfill sites (Pozza et al., 2019).

**Table 1:** Total and size-normalised (< 62.5 µm) metal data for Sydney estuary catchment soils

			Ref			Cd		Co		Cr		Cu		Ni		Pb		Zn
Sydney estuary catchment. Total			1	Mean		0.4		6		19		46		13		194		187
				Median		0.3		5		14		23		8		60		108
*n* = 491				Range		bd-3.4		bd-44		1–265		1–1869		Bd–190		3–9653		4–1807
Sydney estuary catchment. Fine			1	Mean		1.1		16		50		121		33		559		500
				Median		0.9		13		13		60		21		150		259
*n* = 491				Range		bd–8.6		bd–08		7–606		2–5232		bd–440		18–6988		13–6156
Sydney parkland soils. Total			2	Mean		0.4		4		14		38		7		161		174
*n* = 107				Median		0.4		4		12		26		6		121		133
				Range		0.1–1.8		0.7–8		4–48		4–398		1–22		6–1030		21–933
Sydney parkland soils. Fine			2	Mean		1.1		10		36		93		19		388		432
*n* = 107				Median		1.0		10		31		56		16		255		284
				Range		0.4–6		2–29		11–193		24–614		6–86		23–3520		133–3060
Iron cove catchment soils. Total	3	Mean			0.8		5		20		62		12		410		343	
*n* = 374				Median		0.4		4		16		44		10		205		225
				Range		1–24		0.5–24		3–210		5–420		2–65		6–9200		21–2400
Iron cove catchment soils. Fine			3	Mean		2.1		15		56		170		34		1070		927
*n* = 374				Median		1.0		11		43		113		27		501		558
				Range		bd–66		15–69		9–771		21–1500		5–255		39–9573		137–7430
Glebe catchment soils. Total			4	Mean		0.66		NA		NA		114		NA		854		482
*n* = 219				Range		bd-14		NA		NA		2.3–4141		NA		10–0278		20–10254
Homebush Bay catchment. Total			5	Mean		NA		NA		16		33		15		106		155
*n* = 185				Median		NA		NA		14		23		10		67		91
				Range		NA		NA		3–103		4–425		1–202		4–649		bd–5022
Homebush Bay catchment. Fine			5	Mean		NA		NA		32		81		31		227		281
*n* = 185				Median		NA		NA		31		55		21		150		241
				Range		NA		NA		13–60		16–1410		6–1445		23–1302		43–1460
Sydney roadside soil Total *n* = 18			6	Mean		NA		0.18		36		69		147		64		152
Australian garden soil Total			7	Median		2		NA		35		31		2		66		171
*n* = 17,256				Range		< 4–88		NA		< 2–7863		< 1–9157		< 4–657		< 3–2400		< 1–29,400

*NA* Not available, fine ≤ 62.5 µm, *n* = number of samples; *bd * Below detection. References: 1. Birch et al., 2011; 2. Wang et al., 2022; 3. Snowdon & Birch, 2004; 4. Markus and McBratney, 1996; 5. Hodge, 2002; 6. Mohammed et al., 2012; 7. Taylor et al., 2021

An investigation of the whole Sydney estuary catchment (*n* = 491*)* showed soils in the south-eastern region contained the highest metal concentrations (Birch et al., 2011). Soil metal and road network distributions were closely related and results of vehicular emissions modelling, strongly suggested vehicular traffic was the major source of metals to catchment soils (Hodge, 2002; Lawrence, 2006). Soil metals in four Sydney estuary catchment urban parks were also closely related to traffic emissions and adversely affected soil quality and human health (Wang et al., 2022). A study of home gardens showed that Pb was the primary metal of concern and that 40% of the 203 gardens sampled contained soil that exceeded the soil Pb guideline for residential properties (NEPM, 1999) (Rouillon et al., 2017). The highest soil Pb concentrations close to the CBD declined to background levels within 30–40 km of the city centre (Rouillon et al., 2017). In a later nation-wide garden soil survey residential (HIL A; NEPC, 2013) guideline values were exceeded for Pb in 20% of soil samples, but only 4% of samples for Cr and 1% of samples for Cd (Taylor et al., 2021).

Investigations of the relationship between Pb in human blood and catchment soil (Mencel & Thorp, 1976; Garnys et al., 1979; Royal Prince Alfred Hospital and Central and Southern Sydney Area Health Service, 1988; Cooney et al., 1989; Fett et al., 1992; Skinner et al., 1993; Olszowy et al., 1995; Cowie et al., 1997; Markus and McBratney, 2001; Cattle et al., 2002; Gulson et al., 2014) have been comprehensively reviewed by Laidlaw et al. (2017).

## Methods

### Field methods

**Fig. 1:**
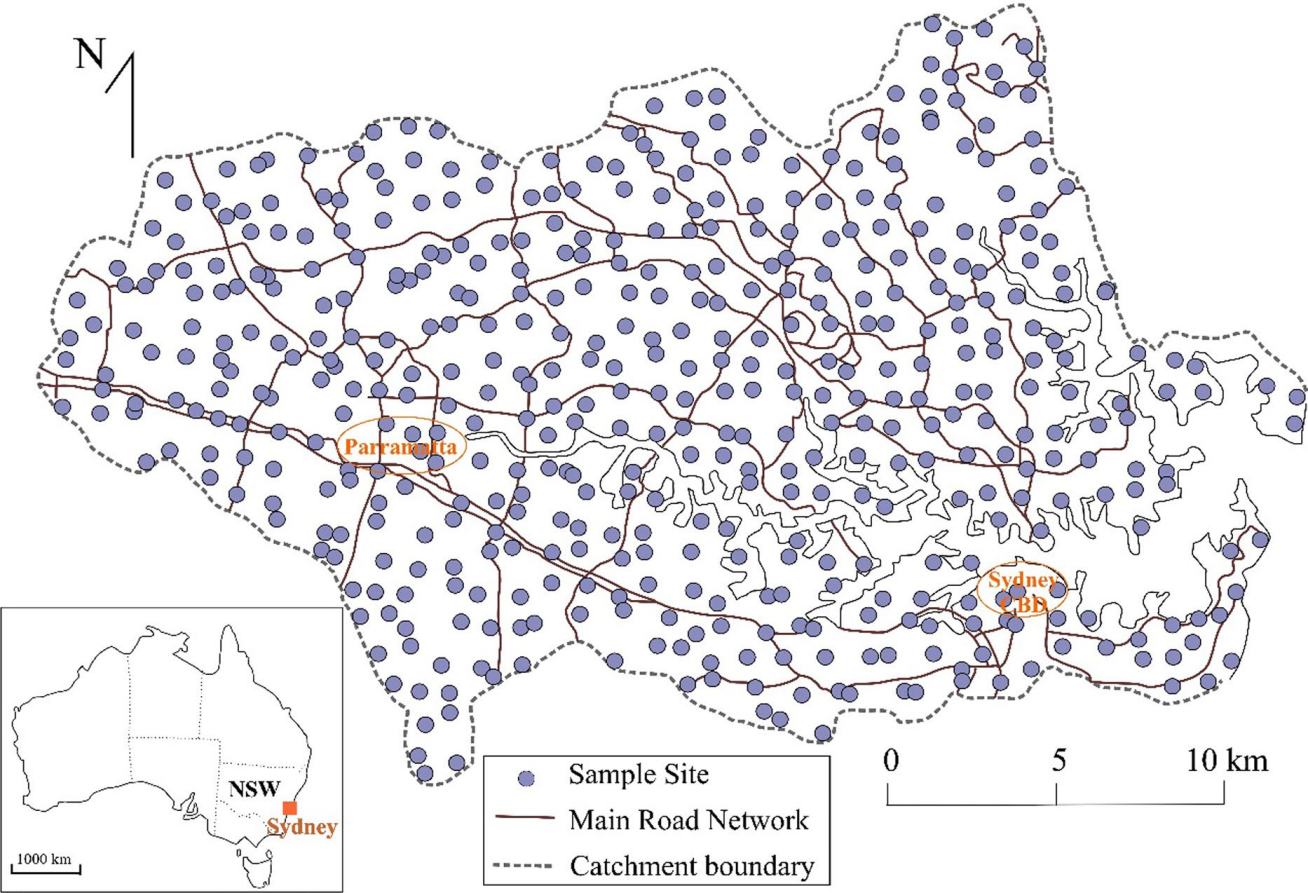
Distribution of soil samples in Sydney estuary catchment (*n* = 491). One sample was taken in each 1 km^2^ in the dominant land use type in that square

The metals data used in the current study were obtained from a previous survey based on samples taken where convenient in the dominant land use type in each one km^2^ gridded area of Sydney estuary catchment (Birch et al., 2011) (Fig. 1). Land uses were residential (45.9%), commercial/industrial (8.1%), road/rail (19.2%), educational/medical (3.4%) and parkland/reserves (23.2%) (Birch & Taylor, 2004; Birch et al., 2015a, b). Four sub-samples collected within 1 m^2^ were pooled to reduce small-scale spatial variability (Birch et al., 2001). Samples were sieved using a 2 mm plastic mesh on site to remove debris and organic fragments and were refrigerated at 4 °C until preparation for analysis.

### Laboratory techniques

Textural variability and heterogeneity of soils was reduced by Post-Extraction Normalisation (PEN) Birch & Taylor, 2000) to identify sources and produce consistent spatial metal distributions (Birch, 2017). The veracity of the PEN method to produce high-quality normalised data was validated by comparison to grain-size (≤ 62.5 µm) and elemental (Fe and Al) normalisation techniques (Birch, 2003; Birch & Snowdon, 2004) and was confirmed by independent assessment (Clark et al., 2000).

In the original investigation (Birch et al., 2011) soils were digested using aqua regia (1:1 HNO_3_:HCl) (modified USEPA 200.8 Rev 4.4 method) and analysed by inductively coupled plasma atomic emission spectrometry (ICP-AES) for seven metals (Cd, Co, Cr, Cu, Ni, Pb and Zn).

### QA/QC

Precision of the analytical method expressed as relative standard deviation (RSD) of replicates (*n* = 26) was < 6%. Accuracy, measured by recovery using a reference material (AGAL-10) (*n* = 28), was between 94 and 104% for all metals. Blanks (*n* = 30) used to measure potential laboratory contamination exhibited negligible metal concentrations.

### Health risk assessment models

The average daily intake (ADI) of a chemical substance through each of three pathways (ingestion ADI_ing_, inhalation ADI_inh_ and dermal contact ADI_dermal_) was determined for children (aged < 2 years) using equations provided by the USEPA (2002) and Bourliva et al. (2018). The values of parameters are presented in Supplementary Table S1.

$${{\text{ADI}}}_{{\text{ing}}}={\text{C}}\times \frac{{{\text{R}}}_{{\text{ing}}}\times {\text{EF}}\times {\text{ED}}}{{\text{BW}}\times {\text{AT}}}\times {10}^{-6}$$

$${{\text{ADI}}}_{{\text{inh}}}={\text{C}}\times \frac{{{\text{R}}}_{{\text{inh}}}\times {\text{EF}}\times {\text{ED}}}{{\text{PEF}}\times {\text{BW}}\times {\text{AT}}}$$

$${{\text{ADI}}}_{{\text{dermal}}}={\text{C}}\times \frac{{\text{SA}}\times {\text{SAF}}\times {\text{ABS}}\times {\text{EF}}\times {\text{ED}}}{{\text{BW}}\times {\text{AT}}}\times {10}^{-6}$$

where R_ing_ = Dust intake rate; R_inh_ = Inhalation rate; EF = Exposure frequency; ED = Exposure duration; BW = Body weight; AT = Average time (year); PEF = Particle emission factor; SA = Exposure skin area; SAF = Skin adherence factor; and ABS = Dermal absorption factor.

The non-carcinogenic Hazard Quotient (*HQ*) of each exposure pathway and Hazard Index (*HI*) were calculated using:

$${HQ}_{i}=\frac{{ADI}_{i}}{{R}_{f}D}$$

$$HI=\sum {HQ}_{i}$$

*HQ*_*i*_ is the non-carcinogenic hazard quotient for each exposure pathway, *R*_*f*_*D* is the reference dose equaling the maximum dose to avoid an adverse reaction when adsorbed, *i* corresponds to the three pathways (Supplementary Material Table S1) and *HI* is the sum of *HQs* for the three exposure pathways. Non-carcinogenic risk occurs when *HI* > 1 and risk increases with the magnitude of *HI* (Doabi et al., 2018).

In the present study, carcinogenic risk (CR) was evaluated by the following equations:

$${CR}_{i}={ADI}_{i}\times SF$$

$${\text{CR}}=\sum {CR}_{i}$$

*SF* (dimensionless) is the carcinogenic slope factor (Supplementary Material Table S2). The acceptable, or tolerable risk for regulatory purposes is 10^−6^ to 10^−4^ to cover for the incremental risk of developing caner during a lifetime of exposure (Ferreira-Baptista et al., 2005).

## Results and discussion

### Total and fine fraction soil metal concentrations

Total soil metal concentrations exhibited considerable variation across the Sydney estuary catchment as was reflected in a wide range in minimum and maximum values for Cu, Pb and Zn. Median concentrations (minimum–maximum) for total Cu, Pb and Zn were 23 (1–1869) µg/g, 60 (3–9653) µg/g and 108 (4–1807) µg/g, respectively, and for normalised concentrations values were 60 (2–5232) µg/g, 150 (18–26,588) µg/g and 259 (13–6156) µg/g, respectively (Birch, et al., 2011) (Table 1). ANZECC/NH&MRC guidelines (ANZECC and NH&MRC, 1992) were exceeded in 17% of samples for Cu, 11% for Pb, and 5% for Zn. The Ecological Investigation Limits (EIL) National Environment Protection Council (2013) recommended values were exceeded in 9%, 6% and 25% of samples, respectively, while Cd, Co, Cr and Ni concentrations were below guidelines (Table 2).

**Table 2:** Total and fine fraction soil metal concentrations and guideline values (µg/g)

*n* = 491	Cd	Co	Cr	Cu	Ni	Pb	Zn	
*Total sediment*								
Minimum	bd	bd	1	1	bd	3	4	
Maximum	3.4	44	265	1869	190	9653	1807	
Mean	0.4	6	19	46	13	194	187	
25 percentile	0.2	2	10	14	5	28	62	
50 percentile	0.3	5	14	23	8	60	108	
95 percentile	1.1	19	44	131	38	723	632	
ANZECC^1^	3	NA	50	60	60	300	200	
% Samples > ANZECC	0.2	NA	3	17	2	11	25	
EIL^2^	3	NA	400	100	60	600	200	
% Samples > EIL	0.2	NA	0	9	2	6	25	
*Fine fraction (< 62.5* µm*)*								
Minimum	bd	bd	7	2	bd	18	13	
Maximum	8.6	108	606	5232	440	26,988	6156	
Mean	1.1	16	50	121	33	559	500	
25 percentile	0.6	8	28	36	14	79	159	
50 percentile	0.9	13	37	60	21	150	259	
95 percentile	2.7	38	120	408	90	1830	1790	

NA  Not available; *bd*  Below detection

^1^Environmental soil quality guidelines (ANZECC and NH&MRC, 1992)

^2^Environmental investigation limits (National Environment Protection Council (2013)

### Sydney catchment soil metal concentration in a global perspective

Metal concentrations in the Sydney estuary catchment soils were higher than reported in most global investigations, e.g. the Danang-Hoian region of Vietnam (Thuy et al., 2000), the city of Xuzhou, China, (Wang & Qin, 2007), Madrid, Spain (De Miguel et al., 1998) and many other global cities, including Seoul, Berlin, Oslo and Glasgow (Table 3). Sydney catchment soils also had substantially higher mean Cu, Pb, and Zn concentrations than average soils for Australia, the European Union and China (Caritat & Cooper, 2011a, b; 2016; Chen et al., 2015; Hu et al., 2020; Tóth et al., 2016) (Table 3).

**Table 3:** Mean metal concentrations for Sydney estuary catchment soils and other global studies (µg/g)

	Refs	Cu	Pb	Zn	
Sydney estuary catchment	1	121	559	500	Fine fraction (< 62.5 µm; normalised)
		46	194	187	Total (< 2 mm)
		60	150	259	50th percentile, normalised
Wollongong City area	2	343	21	82	Total (< 2 mm)
Australian soil average	3	17	11	38	Total (< 2 mm)
Australian continental crust	3	12	7.4	26	Total (< 2 mm)
Global studies					
Seoul, South Korea	4	84	240	271	Total (< 2 mm)
Danang-Hoian Area, Vietnam	5	90	84	153	Total (< 2 mm)
Berlin Metropolitan Area	6	43	78	159	Total (< 2 mm)
Great Britain	7	54	240	260	Total (< 2 mm)
Oslo, Norway	8	32	56	160	< 100 µm; sieve
Xuzhou, China	9	38	43	144	Total (< 500 μm)
Madrid, Spain	10	72	161	210	Total (< 2 mm)
Glasgow, Scotland	11	97	216	207	Total (< 2 mm)
Average European Union soils	12	13	15	na	Total (< 2 mm)
Average Chinese soils	13	27	31	79	Total (< 2 mm)

References: 1. Birch et al., 2011; 2. Beavington, 1973; 3. Caritat & Cooper, 2011a, b, 2016; 4. Chon et al., 1995; 5. Thuy et al., 2000; 6. Birke and Rauch, 1997; 7. Culbard et al., 1988; 8. Tijhuis et al., 2002; 9. Wang & Qin, 2007; 10. De Miguel et al., 1998; 11. Gibson and Farmer, 1985; 12. Tóth et al., 2016; 13. Chen et al., 2015

### Sydney catchment soil metal concentrations and land use

The range of median soil metal concentrations for land use types in Sydney catchment from highest to lowest was: road verge–industrial–commercial–residential–special use–parkland (Table 4). Soil from road verges has frequently been identified with high metal concentrations (Bourliva et al., 2018; Siddiqui et al., 2020; Snowdon & Birch, 2004) with levels declining exponentially with distance from vehicular corridors (Birch & Scollen, 2003; Wang et al., 2022).

**Table 4:** Normalised metal concentrations for land use types

	Residential	Parkland/open space	Road verge	Industrial	Commercial	Special use
*n*	216	98	61	40	40	35
*Copper*						
Minimum	9	2	32	8	19	7
Maximum	332	178	561	5232	4338	131
Mean	72	39	273	291	249	52
25th percentile	38	22	134	64	54	30
50th percentile	58	32	270	90	103	44
95th percentile	170	88	534	551	616	102
*Lead*						
Minimum	29	18	70	43	38	25
Maximum	5569	768	26,988	1795	1577	547
Mean	298	124	2803	304	256	126
25th percentile	86	57	409	99	93	61
50th percentile	157	83	1027	217	161	82
95th percentile	925	286	7900	893	567	362
*Zinc*						
Minimum	60	13	71	76	89	39
Maximum	5679	1162	6156	3556	2747	927
Mean	364	185	1373	780	640	228
25th percentile	172	85	785	271	285	126
50th percentile	238	144	1183	553	495	182
95th percentile	922	404	2630	2347	1974	475

Concentrations in µg/g; *n* = number of samples

A closer examination of NCR posed by Pb in soils from different land uses indicated that 87% of samples recovered from road verges exceeded the guideline for safe exposure (HI > 1) (Table 5). Similarly, 48% of industrial, 33% of residential, and 30% commercial samples exceeded safe Pb levels of exposure for children, but not for adults. In an alternative to using land use type to assess exposure, Garcia-Rico et al. (2016) identified specific areas in Mexico City with the most sensitive populations to exposure (density of children) to produce a ‘sector-based’ assessment called ‘marginality index maps’. In Sydney, Taylor et al., (2021) aggregated risk into ‘significant urban areas’ demarked by Statistical Area Level Boundaries so that risk data could be related to socio-economic indices.

**Table 5:** Number and percentage of samples greater than unity (H > 1) for non-carcinogenic risk for Pb per land use type

	Residential	Parkland/open space	Road verge	Industrial	Commercial	Special use
Number of samples	200	116	61	40	40	35
Number of samples greater than unity	65	5	52	19	12	3
Percentage of samples greater than unity	33	4	87	48	30	9

### Human health CR and NCR assessment

**Fig. 2:**
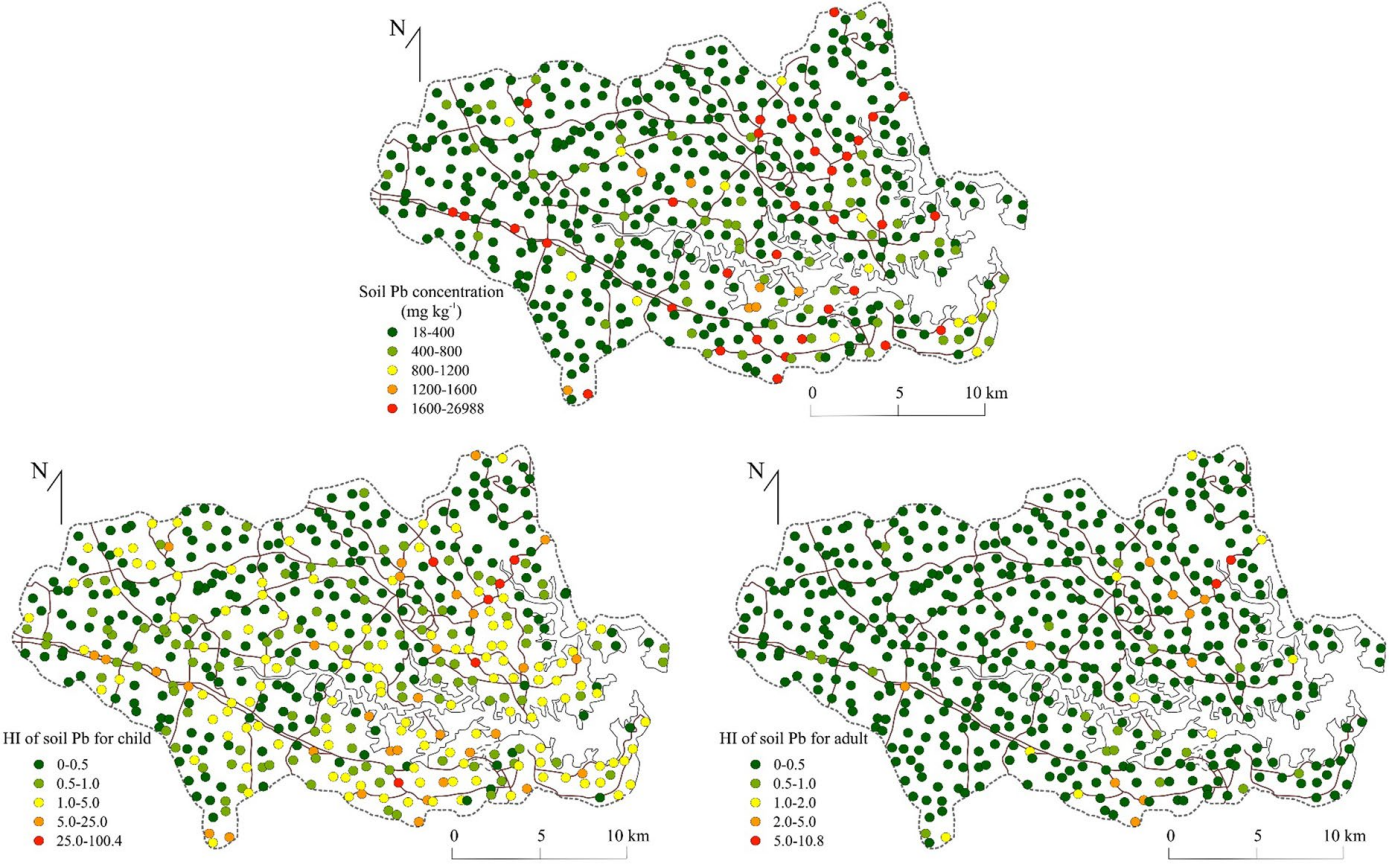
The Pb concentration in catchment soils and the corresponding non-carcinogenic human health risk (HI) for adults and children

**Fig. 3:**
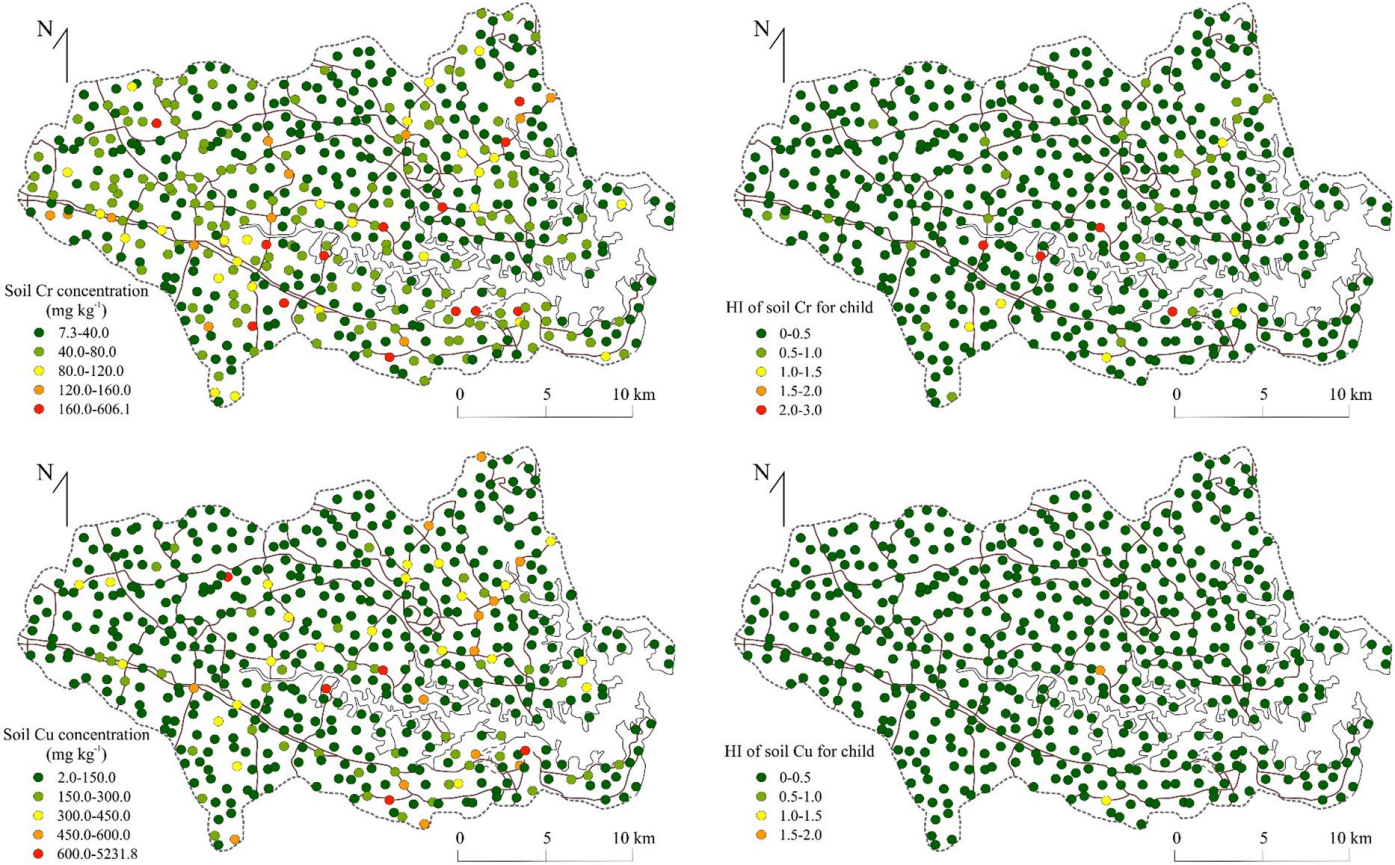
The concentration of Cr and Cu in catchment soils and the corresponding non-carcinogenic human health risk (HI) for children

No soil samples exceeded an HI of unity for Cd, Ni, or Zn and these elements posed no NCR, or CR for children, or adults in Sydney estuary catchment (Table 6). By far the greatest human health risk raised by catchment soils was for Pb, which may pose NCR for children (HI > 1) at 156 sites (32% of samples) and at 21 locations (4.3% of samples) for adults (Fig. 2). Copper at only two sites (0.4% of samples) and Cr at nine locations (1.6% of samples) exceeded unity and may pose minor NCR for children (HI > 1) (Fig. 3). The HQ (individual pathways) and HI (combined pathways) of the remaining studied metals (Cd, Cu, Ni and Zn) were considerably lower than unity, i.e. mean HQ values were < 0.002 and for HI values were < 0.01 (Table 6). Slope factors were only available to calculate CR for Cd, Cr, Pb and Zn (Table 3). No samples exceeded a value of 10^–4^ for Cd indicating no CR and only two samples (0.5%) and one sample exceeded this value for Pb in children and adults, respectively, while only 9 (2%) and 4 (1%) samples exceeded this value for children and adults for Cr, respectively.

**Table 6:** Carcinogenic and non-carcinogenic human health risk for mean and maximum concentrations of Cd, Cr, Cu, Ni, Pb and Zn

	Non-carcinogenic risk (NCR)									
Metal		Concentration (µg/g)	HQ ADIing (child)	HQ ADIing (adult)	HQ ADIinh (child)	HQ ADIinh (adult)	HQ ADIdermal (child)	HQ ADIdermal (adult)	HI child	HI adult
Cd	Mean	1.1	1.41E−02	1.51E−03	3.93E−07	2.21E−07	3.94E−03	6.01E−04	1.80E−02	2.11E−03
	Max	8.6	1.10E−01	1.18E−02	3.08E−06	1.74E−06	3.09E−02	4.72E−03	1.41E−01	1.65E−02
Cr	Mean	50	2.13E−01	2.28E−02	6.24E−04	3.52E−04	2.98E−02	4.55E−03	2.43E−01	2.77E−02
	Max	606	2.58E+00	2.77E−01	7.57E−03	4.27E−03	3.62E−01	5.52E−02	*2.95E*+*00*	3.36E−01
Cu	Mean	122	3.91E−02	4.19E−03	1.09E−06	6.13E−07	3.65E−04	5.58E−05	3.95E−02	4.25E−03
	Max	5232	1.67E+00	1.79E−01	4.65E−05	2.62E−05	1.56E−02	2.38E−03	*1.69E*+*00*	1.82E−01
Ni	Mean	33	2.11E−02	2.26E−03	5.72E−07	3.22E−07	2.19E−04	3.34E−05	2.13E−02	2.29E−03
	Max	440	2.81E−01	3.02E−02	7.63E−06	4.31E−06	2.92E−03	4.46E−04	2.84E−01	3.06E−02
Pb	Mean	579	2.12E+00	2.27E−01	5.88E−05	3.32E−05	3.95E−02	6.03E−03	*2.16E*+*00*	2.33E−01
	Max	26,988	9.86E+01	1.06E+01	2.74E−03	1.54E−03	1.84E+00	2.81E−01	*1.00E*+*02*	*1.08E*+*01*
Zn	Mean	508	2.17E−02	2.32E−03	6.05E−07	3.41E−07	3.03E−04	4.63E−05	2.20E−02	2.37E−03
	Max	6156	2.62E−01	2.81E−02	7.33E−06	4.13E−06	3.67E−03	5.61E−04	2.66E−01	2.87E−02

	Carcinogenic risk (CR)																			
Metal			Concentration (µg/g)		CRing (child)			CRing (adult)		CRinh (child)		CRinh (adult)		CRdermal (child)		CRdermal (adult)		CR child		CR adult
Cd		Mean		1.1		7.40E−06	3.17E−06		2.13E−10		4.81E−10		2.07E−08		1.26E−08		7.42E−06		3.18E−06	
		Max		8.6		5.77E−05	2.47E−05		1.67E−09		3.76E−09		1.62E−07		9.87E−08		5.79E−05		2.48E−05	
Cr		Mean		50		2.74E−05	1.18E−05		6.42E−08		1.45E−07		3.07E−06		1.87E−06		3.06E−05		1.38E−05	
		Max		606		3.33E−04	1.43E−04		7.79E−07		1.76E−06		3.72E−05		2.27E−05		**3.71E**−**04**		**1.67E**−**04**	
Cu		Mean		122		NA	NA		NA		NA		NA		NA		NA		NA	
		Max		5232		NA	NA		NA		NA		NA		NA		NA		NA	
Ni		Mean		33		NA	NA		8.49E−10		1.92E−09		NA		NA		8.49E−10		1.92E−09	
		Max		440		NA	NA		1.13E−08		2.55E−08		NA		NA		1.13E−08		2.55E−08	
Pb		Mean		579		5.21E−06	2.23E−06		7.19E−10		1.62E−09		2.92E−08		1.78E−08		5.24E−06		2.25E−06	
		Max		26,988		2.51E−04	1.08E−04		3.47E−08		7.83E−08		1.41E−06		8.60E−07		**2.53E**−**04**		**1.09E**−**04**	
Zn		Mean		508		NA	NA		NA		NA		NA		NA		NA		NA	
		Max		6156		NA	NA		NA		NA		NA		NA		NA		NA	

*HQ* Hazard quotient; *ADI* Average daily intake; *ing* Ingestion; *inh* Inhalation; *HI* Hazard index

*NA* Not available; Italics indicates HI > 1; Bold indicates CR > 10^–4^

NCR was more prevalent than CR in most global studies. In China, although CR and NCR was low for soils in most areas (Wang et al., 2019), Hu et al. (2020) identified a potential CR and NCR threat for children in some provinces, but not for adults. Human activities considerably contributed toxic elements to agricultural soils in the Frydek Mistek district of Czech Republic and some CR hotspots were recommended for immediate remediation (Agyeman et al., 2021). CR was only evident for Cr in urban dust in commercial land use areas in Tehran (Mihankhan et al., 2020), while in Mexico City Pb in road dust posed a NCR and As and Cr presented a CR (Garcia-Rico et al., 2016). NCR was highest for Cr and Pb in road dust from Thessaloniki city, Greece, while CR was not a threat for Cd, Cr, or Ni (Bourliva et al., 2018).

### Exposure for adults and children

In the current study, NCR and CR were consistently more likely for children than for adults, which was typical for other countries, e.g. in China (Chen et al., 2015; Hou et al., 2019; Hu et al., 2020) and Delhi (Roy et al., 2019; Siddiqui et al., 2020). Approximately 6.1% of the Frydek Mistek district of Czech Republic posed a potential NCR to children rather than to adults and 13.05% of the sampled locations were carcinogenic to children (Agyeman et al., 2921). Children were also more at risk of carcinogenic disease in Thessaloniki city, Greece (Bourliva et al., 2018) and in Angola Ferreita-Baptista and De Miguel (2005). The combined Hazard Index (HI) for children through different routes of exposure was 8.9 times greater than for adults in Karachi city and CR from Pb due to oral ingestion of soil exceeded a value of 1 × 10^−6^ in some areas of the city for children (Karim & Qureshi, 2014). Children are more suseptable to CR and NCR due to oral hand and finger activities with exposure through ingestion.

### Metals associated with human health risk

In the current investigation, six metals (Cd, Cr, Cu, Ni, Pb and Zn) were assessed for CR and NCR; however, only Cr and Pb posed minor CR, and NCR was only prevalent for Pb with Cr and Cu raising minor NCR risk. Soil metals assessed for human health risk in global studies were highly variable and included As, Cd, Co, Cr, Cu, Fe, Hg, Mn, Ni, Pb and Zn. However, only a small number of these elements have been proved to pose a health risk and these varied greatly between investigations. In China, Cd and Hg were most likely to pose NCR (Hu et al., 2020) with As, Pb, Cr and Ni exerting a lower risk (Chen et al., 2015). Metals in urban soils in Shenyang City (NE China) posed low health risk, while As showed the highest CR and Cr and Pb raised a significant NCR (Wang et al., 2019). Chrome also had a strong CR risk for adults through inhalation in soil from potato-producing areas in Guizhou Province, China.

Studies of road dust, a common secondary source of metals to road-side soils, also revealed variable metals associated with a health risk. A nation-wide study (*n* = 3877) of road dust in China, found Pb posed the highest health risk (Hou et al., 2019), while road dust from areas not affected by exhaust traffic emissions (trunk roads) were within safe limits for all assessed metals (Liu et al., 2014). Chromium and Pb in road dust from Thessaloniki city, Greece were rated with the highest NCR (Bourliva et al., 2018), whereas Co, Cu, Fe, Mn, and Zn were safe in road dust and soils in Delhi (Roy et al., 2019), but not for Ni, which may pose a CR in urban areas (Siddiqui et al., 2020).

### Exposure pathways for NCR and CR

In the present study, the main exposure pathway driving NCR for all metals was ingestion, which accounted for most of the risk (> 95%), while dermal (< 5%) and inhalation (< 1%) were of minor risk. Uptake for CR was almost equally distributed between the three exposure pathways and exposure was considerably greater (approximately 10 times for all metals) for children than adults for all metals analysed.

Ingestion was the most important pathway in most global studies with either dermal, or inhalation pathways being next important, e.g. in NE China (Hou et al., 2019; Wang et al., 2019). Ingestion was also the main risk for NCR for children in Mexico City (Garcia-Rico et al., 2016) and CR was high for all possible pathways in Delhi, especially for Ni in roadside soils and road dust (Siddiqui et al., 2020). The importance of pathways ranked ingestion > dermal > inhalation in road dust from 10 districts in Thessaloniki city, Greece (Bourliva et al., 2018). Although ingestion was most prevelent pathway noted in the literature, soil Cr and As from potato-producing areas in Guizhou Province, China, were predicted to produce CR through dermal contact.

### Source of soil metals posing a human health risk

A detailed study of soil in four recreational parks in Sydney showed Cu, Pb and Zn were related to traffic emissions and traffic volumes (Wang et al., 2022). Metal concentrations in park soils were highly elevated and concentrations decreased exponentially with distance from arterial roads. Traffic emission accounted for 72–84% of metal contamination in soils of parks surrounded by high traffic volumes, whereas emission values were 25–70% for park soil with no surrounding arterial road network. Copper and Zn in soils of the four parks contributed no NCR for children, or adults and Pb had negligible health risk for adults, however Pb in soil in parks near arterial roads, may raise NCR for children due to traffic emissions (Wang et al., 2022).

Roadside soil and street dust are frequently identified as media associated with high metal concentrations (Birch & Scollen, 2003; Ferreita-Baptista and De Miguel, 2005; Birch et al., 2011; Bourliva et al., 2018; Mihankhan et al., 2020). An early study reported 40% of the total 17,000 t of particulates emitted to the Sydney atmosphere annually were from motor vehicles (Forrest, 1991) and an atmospheric deposition model of vehicular emissions showed a close relationship between road density and atmospheric metal concentration for the catchment (Lawrence, 2006). Atmospheric deposition of pollutants in metropolitan Sydney accounted for approximately 50% of the total Cu, Pb and Zn deposition in the vicinity of minor roads (Davis & Birch, 2011). Leaded petrol emitted approximately 68,000 t of Pb into the atmosphere in New South Wales (NSW) between 1958 and 2002 (Kristensen, 2015) and leaded gasoline contributed approximately 90% of Pb to Sydney air between 1980 and 2001 (Chiaradia et al., 1997).

A detailed study of road surfaces in Sydney showed the mass of material accumulating on roads was substantial and closely related to vehicle density (Birch & Scollen, 2003). Road-derived material is highly resuspendable with 34%, 43% and 33% of the Cu, Pb and Zn being in the < 63 µm fraction and 90%, 91% and 89% being < 200 µm, respectively. This observation is similar to results obtained from a London street where 62% of total Pb and similar proportions of Cu and Zn were associated with the 100–500 µm fraction (Ellis & Revitt, 1982; Revitt et al., 1990). The fine-grained nature of this material renders it susceptible to resuspension and uptake by inhalation and ingestion increasing CR and NCR, High-density sampling showed consistently elevated Cu, Pb and Zn concentrations for roadside soils and road dust across Sydney catchment (Hodge, 2002; Snowden and Birch, 2004; Birch & McCready, 2009; Birch, 2011) and of the 138 road-related samples available, 38% posed a NCR by Pb for children.

Global investigations of road-derived materials also revealed variable metals associated with a health risk. A nation-wide study (*n* = 3877) of road dust in China, showed Pb posed the highest health risk and was the only metal with an HQ > 1 (Hou et al., 2019). The source of the Pb was mainly traffic and industrial activities (Hou et al., 2019). In Thessaloniki city, Greece, Cr and Pb rated the highest NCR in road dust (Bourliva et al., 2018), whereas in Mexico City, street dust posed the highest health risk for all metals (Garcia-Rico et al., 2016).

These studies emphasise the considerable influence traffic emissions have on metal concentrations of urban soil, which can confidently be predicted to have implications for human health (Wang et al., 2022).

### Limitations of CR and NCR assessments and recommendations

Biometric exposure parameters for different populations vary greatly by race, region and habitat and existing parameters, e.g. weight, height and breathing volumes/adsorption rate may not represent exposure for the community being assessed (Ferreita-Baptista and De Miguel, 2005; Hou et al., 2019). Soil screening guides (US EPA, 1996a, b) and exposure models (US EPA, 2002) are two decades old and may require revision to service a wider population. Metals and organic pollutants in urban soil for which data are unavailable may affect human health, but are not considered in assessments and therefore risk may well be higher than typically calculated (Drage et al., 2015).

Fine-grain sizes are commonly used in soil health assessments on the assumption that smaller particles have greater adsorption, resuspension and aeolian transport potential. However, the size of the material being analysed for metals is also critical in determining element concentrations (Forstner, 1982; Forstner & Wittmann, 1979). For example, the concentrations of metals in > 200 µm road-side material from Sydney catchment were 87 µg Cu/g, 202 µg Pb/g and 259 µg Zn/g, respectively, compared to 187 µg Cu/g, 723 µg Pb/g and 1240 µg Zn/g for the < 62.5 µm fraction (Birch & Scollen, 2003). The size of material being used for human health assessment globally varies substantially, e. g. the size of material analysed from playgrounds, roofs and roads in Mexico City was < 44 µm (Garcia-Rico et al., 2016) and < 63 µm-sized particles were analysed in Delhi for soils and road dust (Siddiqui et al., 2020). Bourliva et al. (2018) analysed 500 µm material in Thessaloniki city, Greece and street dust between 63 and 500 µm was analysed in an assessment of human health risk in dust from 53 cities (*n* = 3877) across China (Hou et al., 2019). Soil was simply ‘sieved’ in China (Chen et al. 2016), while Wang et al., (2019) sieved urban topsoil at 2 mm in Shenyangn City, China. Ferreita-Baptista and De Miguel (2005) quoted analysed material sizes between 2000 and 63 µm for 15 studies, including one investigation using < 595 µm-sized particles. Not only will analyses of these materials produce highly varied metal concentrations due to particle size alone, different sized particles will have varied transport and adsorption potential and will produce incomparable results.

Sedimentary metals are present in the mineral matrix and as the absorbed phase of fine-grained particles (Ackermann et al., 1983; Forstner, 1982). Only chemicals available through the three exposure pathways should be included in analysis of soils and road-derived material used for human health studies. Metals incorporated tightly within the mineral matrix, some of which are present in high concentrations, should be excluded from the assessment. Analytical methods that assess both the matrix and absorbed phases will confound interpretation of human health risk and thus the digestion method used to analyse metals in soils is of fundamental importance in assessment of NCR and CR (Birch et al., 2020). Strong acid digestions, e.g. HF break down minerals and release both matrix and adsorbed components resulting in a 4- to ninefold elevation of metal concentrations compared to weaker acids (e.g. frequently used aqua regia), whereas more diluted acid solutions recover < 60% of metals relative to aqua regia (Katz & Kaplan, 1981). Digestion procedures used in assessment of human health reported in the literature were highly varied and included a large number of acids from weak to strong, e.g. HNO_3_; HCl + HNO_3_; HNO_3_ + HClO_4_; HNO_3_ + HClO_4_ + HF; HF + HClO_4_. This large range of acids used in digestion will result in a mixed proportion of matrix and adsorbed metals being analysed, resulting in highly varied risk profiles and reduced comparability between studies.

The use of inconsistent soil size, metal species and digestion techniques will result in incomparable CR and NCR assessment. The < 63 µm fraction is easily fractionated from the total soil, is readily resuspendable, has high adsorption potential, is the most frequently used size in environmental procedures and should be considered as the routine size fraction for human health risk assessment. Acids and acid mixtures, which remove only the adsorbed phase, should be used for digesting soils for CR and NCR assessment, e.g., HCl and HCl:HNO_3_.

## Conclusions

Soil metal concentrations in Sydney estuary catchment were generally higher than observed in most global capital cities and soil guidelines were exceeded for Cu, Pb and Zn. The greatest concentration of metals in the catchment was for gully pots and road dust and roadside soil metals decreased exponentially with distance from vehicular corridors. This evidence strongly suggested that the source of metals was related to traffic density and historical use of Pb additives in petrol.

Soil Cd, Ni and Zn posed no NCR, or CR for children, or adults in Sydney estuary catchment, while Cu and Cr may pose minor NCR for children. The greatest human health risk raised by catchment soils was from Pb, which may pose NCR for children and adults. For CR and NCR determinations to be comparable, chemical and physical techniques used in assessment need to be consistent and standardised.

### Supplementary Information

Below is the link to the electronic supplementary material.

Supplementary file1 (DOCX 19 kb)

## Data Availability

Data used in the present work are available on request.
