# Supplementary material for: Human health risk assessment of metal-contaminated soils in Sydney estuary catchment (Australia)
Source: Environ Geochem Health. 2024 Mar 14;46(4):125. doi: 10.1007/s10653-024-01898-4 (PMC10940391; doi:10.1007/s10653-024-01898-4)
Supplement: Supplementary file 1 — Supplementary file1 (DOCX 19 kb) [file 10653_2024_1898_MOESM1_ESM.docx]

Supplementary Material

Environmental Geochemistry and Health; Authors: Gavin Birch^a*^, Xiaoyu Wang^b^ and Enfeng Liu^b^; affiliations: ^a^ Geocoastal Research Group, School of Geosciences, Sydney University, Sydney, NSW, 2006, Australia. ^b^ College of Geography and Environment, Shandong Normal University, Ji’nan, 250358, PR China.

* Corresponding author.

gavin.birch@sydney.edu.au

Table S1 The names and values of parameters included in the human health risk assessment model.

|  | Meaning | Value | | References |
| --- | --- | --- | --- | --- |
|  |  | Child | Adult |  |
| *R_ing_* | The dust intake rate  (mg day^-1^) | 200 | 100 | (USEPA, 2002) |
| *R_inh_* | The inhalation rate  (m^3^ day^-1^) | 7.6 | 20 | (Ferreira-Baptista et al., 2005) |
| *EF* | The exposure frequency  (day year^-1^) | 350 | | (USEPA, 2002) |
| *ED* | The exposure duration  (year) | 6 | 24 | (Bourliva et al., 2018) |
| *PEF* | The particle emission factor (m^3^ kg^-1^) | 1.36×10^9^ | | (USEPA, 2002) |
| *SA* | The exposure skin area (cm^2^ day^-1^) | 2800 | 5700 | (USEPA, 2002) |
| *SAF* | The skin adherence factor (mg cm^-2^) | 0.2 | 0.07 | (USEPA, 2002) |
| *ABS* | The dermal absorption factor | 0.001(As:0.03) | | (Ferreira-Baptista et al., 2005) |
| *BW* | The body weight (kg) | 15 | 70 | (USEPA, 2002) |
| *AT* | The average time (year) | *ED*×365 days for non-carcinogens and 365*70 for carcinogens; | | (Bourliva et al., 2018) |

References: Bourliva et al., 2018; Doabi et al., 2018; Ferreira-Baptista, L., De Miguel, E., 2005; US EPA, 2002

Table S2 The reference dose for the three exposure pathways and carcinogenic slope factor (SF) for each metal.

|  |  |  |  |  |  |  |  |  |
| --- | --- | --- | --- | --- | --- | --- | --- | --- |
|  | Cd | Cr | Cu | Ni | Pb | Zn |  |  |
| *R_f_D_ing_* | 1.00E-03 | 3.00E-03 | 4.00E-02 | 2.00E-02 | 3.50E-03 | 3.00E-01 |  |  |
| *R_f_D_inh_* | 1.00E-03 | 2.86E-05 | 4.02E-02 | 2.06E-02 | 3.52E-03 | 3.00E-01 |  |  |
| *R_f_D_dermal_* | 1.00E-05 | 6.00E-05 | 1.20E-02 | 5.40E-03 | 5.25E-04 | 6.00E-02 |  |  |
| *SF_ing_* | 6.10E+00 | 5.01E-01 |  |  | 8.50E-03 |  |  |  |
| *SF_inh_* | 6.30E+00 | 4.20E+01 |  | 8.40E-01 | 4.20E-02 |  |  |  |
| *SF_dermal_* | 6.10E+00 | 2.00E+01 |  |  | 1.70E-02 |  |  |  |
|  | | | | | | | | |

References: Bourliva et al., 2018; Doabi et al., 2018; Ferreira-Baptista, L., De Miguel, E., 2005; US EPA, 2002; Wang et al., 2019.
